# Nafamostat mesylate versus low-molecular-weight heparin during continuous renal replacement therapy in critically Ill adults: a single-center retrospective cohort study

**DOI:** 10.1186/s12882-026-05351-9

**Published:** 2026-09-07

**Authors:** Xianguo Zeng, Yutong Xiao, Chaoqun Zhang, Yide Li, Yongjun Liu, Liang Luo

**Affiliations:** 1 Department of Critical Care Medicine, The Seventh Affiliated Hospital of Sun Yat-sen University, No. 628 Zhenyuan Road, Shenzhen, Guangdong 518107 China; 2 Department of Critical Care Medicine, The First Affiliated Hospital of Sun Yat-sen University, No. 58 Zhongshan Er Road, Guangzhou, Guangdong 510080 China; 3 Guangdong Clinical Research Center for Critical Care Medicine, Guangzhou, Guangdong China

**Keywords:** Anticoagulation, Continuous renal replacement therapy, Filter survival, Low-molecular-weight heparin, Nafamostat mesylate

## Abstract

**Background:**

Nafamostat mesylate (NM) is frequently used for continuous renal replacement therapy (CRRT) anticoagulation when clinicians wish to avoid sustained systemic anticoagulation, whereas low-molecular-weight heparin (LMWH) is commonly selected when systemic anticoagulation is considered acceptable. However, direct evidence comparing NM with LMWH in adult intensive care unit (ICU) patients undergoing CRRT remains limited. This study compared filter lifespan, circuit-related failure and safety outcomes between NM and LMWH in a real-world ICU CRRT cohort.

**Methods:**

This single-center retrospective cohort study included adult ICU patients receiving CRRT with NM or LMWH between January 2022 and January 2023. The primary outcome was filter lifespan. In the primary time-to-event analysis, circuit-related filter failure was defined as clotting-related failure or access/device-related failure, while planned and clinical-event-related terminations were censored. Kaplan–Meier analysis, Cox regression with patient-level cluster-robust standard errors, shared-frailty Cox regression, and propensity score–matched analyses were used to compare filter survival, account for repeated circuits within patients, adjust for CRRT prescription factors, and assess robustness.

**Results:**

A total of 116 patients contributing 344 filters were included: 31 patients with 132 filters in the NM group and 85 patients with 212 filters in the LMWH group. NM-treated patients had greater illness severity and more pronounced coagulation abnormalities. Unadjusted Kaplan–Meier analysis showed shorter filter survival with NM than with LMWH (log-rank *P* = 0.007). However, anticoagulant strategy was not significantly associated with circuit-related filter failure in the primary Cox model (HR 1.41; 95% CI 0.88–2.25; *P* = 0.15), shared-frailty Cox model (HR 1.57; 95% CI 0.85–2.90; *P* = 0.15), extended Cox model (HR 1.16; 95% CI 0.70–1.90; *P* = 0.56), or propensity score–matched analyses. Bleeding events were infrequent, precluding a precise comparison of bleeding risk between groups.

**Conclusions:**

Anticoagulant strategy was not significantly associated with circuit-related filter failure after adjustment for measured covariates. Because of substantial baseline differences, residual confounding, and limited statistical precision, these findings should not be interpreted as evidence of comparative efficacy, equivalence, noninferiority, or comparable safety. Larger prospective studies with standardized treatment protocols are needed.

**Supplementary Information:**

The online version contains supplementary material available at https://doi.org/10.1186/s12882-026-05351-9.

## Background

Continuous renal replacement therapy (CRRT) is the preferred renal support modality for critically ill patients with acute kidney injury (AKI) [1], particularly in those with hemodynamic instability or complex fluid and metabolic disturbances. Maintaining extracorporeal circuit patency remains a major challenge during CRRT because hemoconcentration and contact between blood and artificial surfaces promote coagulation activation and circuit clotting [2]. Premature circuit failure may reduce delivered dialysis dose, increase treatment downtime and blood loss, and increase workload and cost [3, 4]. Therefore, anticoagulation during CRRT must balance effective circuit patency against the risk of bleeding [5]. 

Low-molecular-weight heparin (LMWH) is used in a substantial minority of patients during CRRT [6]. Potential advantages of LMWH include convenient administration, a predictable anticoagulant effect, and a lower risk of heparin-induced thrombocytopenia compared with unfractionated heparin (UFH) [7, 8]. However, LMWH may increase bleeding risk [9] and accumulate in patients with renal dysfunction [10], requiring dose adjustment and careful monitoring. Several studies have compared LMWH with regional citrate anticoagulation (RCA) [11–14] or UFH [15], with variable efficacy and safety findings.

Nafamostat mesylate (NM), a synthetic serine protease inhibitor [16] with a very short half–life [17], is commonly used in East Asian countries [18] as an alternative anticoagulant for extracorporeal therapies. Because of its short systemic exposure, NM is often selected when clinicians wish to avoid sustained systemic anticoagulation. Previous studies have compared NM with UFH [19], no anticoagulation [20, 21], or RCA [22], whereas direct evidence comparing NM with LMWH in adult CRRT populations remains limited.

This evidence gap is clinically relevant because NM and LMWH are both used in routine CRRT practice, but often in different clinical contexts. LMWH is generally selected when systemic anticoagulation is considered acceptable, whereas NM is frequently chosen when bleeding risk is perceived to be high. This creates substantial confounding by indication, particularly because “high bleeding risk” lacks a universally accepted definition in critically ill patients undergoing CRRT; [23] patients considered at high bleeding risk in one clinical setting may not be classified similarly in another.

Interpreting filter survival in this setting is complex. Filter survival may reflect anticoagulant-related effects, but it may also be influenced by patient selection, vascular access, CRRT modality, dilution strategy, filtration fraction, filter type and repeated circuit use within the same patient. Accordingly, comparative studies of CRRT anticoagulation should consider patient-level differences, within-patient clustering from repeated circuits, and circuit-level prescription factors. In this single-center retrospective cohort study, we compared NM and LMWH anticoagulation in adult ICU patients receiving CRRT, focusing on filter lifespan, circuit-related failure and safety outcomes, and examined whether observed differences persisted across analyses accounting for circuit-level prescription factors and measured patient-level differences.

## Methods

### Study design and participants

This single-center retrospective cohort study was conducted in the intensive care unit (ICU) of the First Affiliated Hospital of Sun Yat-sen University. All CRRT treatments delivered between January 1, 2022 and January 31, 2023 were screened through the institutional Health Information System and ICU monitoring database. Adults aged ≥ 18 years who received at least one CRRT circuit and remained in the ICU for > 24 h were eligible.

Patients were excluded if they received therapeutic-dose anticoagulation or antiplatelet therapy; used anticoagulation strategies other than NM or LMWH within a given circuit; switched anticoagulant regimen before circuit termination; had documented hypersensitivity to NM or LMWH; received CRRT through an arteriovenous fistula; underwent concurrent extracorporeal therapies, including extracorporeal membrane oxygenation, intra-aortic balloon pump, plasma exchange or bilirubin adsorption; or were pregnant or breastfeeding. Circuits disconnected and left idle during surgery or diagnostic examinations were excluded because idle circuit time could artificially influence filter lifespan. Patients who initially received CRRT without anticoagulation but subsequently started NM or LMWH after circuit replacement remained eligible. No between-circuit crossover between NM and LMWH occurred. To reduce selection and information bias, all eligible patients and circuits during the 13-month study period were consecutively included, and data were independently extracted by two researchers using a standardized data extraction form. Discrepancies were resolved by review and discussion. Missing data were minimal and limited to height or weight; these values were obtained from prior hospitalization records or other existing clinical documentation. After retrieval, no missing data remained for variables included in the analyses. The primary clinical basis for CRRT initiation was classified using existing clinical records. The category “KDIGO stage 3 with oliguria/anuria and progressive azotemia” denoted patients with documented KDIGO stage 3 acute kidney injury before CRRT initiation, in whom oliguria or anuria accompanied by progressive azotemia formed the principal clinical basis for continuous therapy. “Maintenance dialysis–dependent kidney failure” denoted patients receiving maintenance dialysis before ICU admission who required CRRT during critical illness. Although the NM cohort overlaps with that used in our previously published NM–RCA study [22], the present study addresses a distinct comparator and clinical question; all analyses were newly conducted, and no numerical results, tables, figures or text were reused. The study was reported in accordance with the Strengthening the Reporting of Observational Studies in Epidemiology (STROBE) statement [24]. 

### Anticoagulation and continuous renal replacement therapy protocols

Anticoagulant selection was not governed by a prespecified institutional algorithm, a formal bleeding-risk score, or fixed laboratory thresholds. The choice between NM and LMWH was individualized by the treating intensivists according to the overall bedside clinical assessment. Routine bleeding-risk assessment considered thrombocytopenia (particularly platelet count < 100 × 10⁹/L), markedly prolonged coagulation times (e.g., aPTT > 60 s or INR > 2.0), active hemorrhage, recent surgery (particularly surgery within 48 h or major surgery within 7 days), recent cerebral hemorrhage or a history of major cerebral bleeding, and clinical conditions associated with coagulopathy, such as septic shock or DIC. These features were considered collectively rather than applied as mandatory criteria for anticoagulant allocation. In routine practice, LMWH was generally avoided in patients with active bleeding, whereas NM was commonly selected in patients with liver failure accompanied by prolonged coagulation times and/or bleeding, or when sustained systemic anticoagulation was otherwise considered undesirable. The patient-specific rationale for anticoagulant selection and clinician-defined bleeding risk were not recorded in standardized data fields and could not be reliably reconstructed for every patient.

Vascular access was established through the femoral or internal jugular vein using 12-Fr double-lumen catheters. CRRT was performed using Gambro or Fresenius platforms with AV1000S, ST150, M150, or oXiris filters. oXiris was generally selected when adsorption was clinically desired, most commonly in patients with sepsis or marked systemic inflammation. NM circuits were primed with 20 mg NM dissolved in 5% dextrose and added to 500 mL normal saline. NM was infused at 2–40 mg/h and titrated to a post-filter activated clotting time of 160–200 s. All LMWH-treated circuits received enoxaparin sodium. Dosing followed the Chinese Standard Operating Procedures for Blood Purification (2021 edition) [25], with an initial intravenous bolus of 60–80 IU/kg followed by 30–40 IU/kg every 4–6 h and progressive dose reduction during prolonged CRRT according to coagulation parameters and clinical response.

CRRT modality, dilution strategy, filter type, and blood flow rate were selected by the treating physicians. Blood flow was generally maintained at 100–220 mL/min. Filtration fraction was calculated as the ultrafiltration flow entering the hemofilter divided by plasma water flow; for pre-dilution circuits, the prefilter replacement-fluid flow was incorporated into the effective blood flow entering the filter. For CVVHD circuits without convective replacement, filtration fraction was considered not applicable and treated as approximately zero.

### Outcomes

The primary outcome was filter lifespan, defined as the elapsed time from initiation to termination of each CRRT circuit. Reasons for circuit termination were classified into four mutually exclusive categories: planned termination, clotting-related failure, access/device-related failure, and clinical-event-related termination. Clotting-related failure referred to circuit termination because of visible filter or circuit clotting. Access/device-related failure included catheter kinking or malposition, inadequate blood flow, catheter dysfunction, high venous pressure, elevated or low transmembrane pressure, and other mechanical or device-related pressure abnormalities. Clinical-event-related termination included termination for surgery, diagnostic procedures, discharge, withdrawal of care, cardiac arrest, or death.

For the primary time-to-event analysis, circuit-related filter failure was defined as clotting-related failure or access/device-related failure. Planned termination and clinical-event-related termination were treated as censored observations because these events did not necessarily indicate loss of circuit patency. For all included circuits, filter lifespan was calculated as the elapsed clock time from circuit initiation to final termination. Temporary interruptions that did not result in definitive circuit termination, including brief periods of circuit recirculation, were retained as part of the same circuit episode and were not subtracted from filter lifespan. Circuits electively discontinued for a procedure or imaging study were classified according to the recorded termination reason, whereas circuits left idle during surgery or examination were excluded during cohort construction. Two alternative event definitions were used for sensitivity analyses. In the clot-specific failure model, only clotting-related termination was considered an event. In the non-planned termination model, all non-planned terminations were considered events.

Secondary outcomes included ICU and hospital length of stay, in-hospital mortality, 28-day mortality, clinically documented bleeding events, red blood cell (RBC) transfusion, acid-base or electrolyte disturbances and allergic reactions. Bleeding events were defined as clinically documented bleeding requiring transfusion of ≥ 2 units of RBCs during CRRT or procedural or surgical hemostasis. RBC transfusion was analyzed separately and defined as receipt of any RBC transfusion during CRRT.

Adverse clinical events were assessed during CRRT. Metabolic acidosis was defined as pH < 7.20 with bicarbonate < 20 mmol/L when not primarily attributable to lactic acidosis. Severe metabolic alkalosis was defined as pH > 7.50 with bicarbonate > 30 mmol/L. Hyperkalemia and hypokalemia were defined as potassium > 5.4 mmol/L and < 3.5 mmol/L, respectively. Hypercalcemia was defined as ionized calcium > 1.4 mmol/L, and severe hypocalcemia as ionized calcium < 0.9 mmol/L. Allergic reactions were defined as symptoms documented in medical or nursing records and considered temporally related to anticoagulant use, including rash, pruritus or unexplained hypotension.

### Statistical analysis

Continuous variables were summarized as mean ± standard deviation or median with interquartile range, and categorical variables as counts and percentages. Between-group comparisons used Student’s t-test or Mann–Whitney U test for continuous variables and the chi-square test or Fisher’s exact test for categorical variables, as appropriate. Filter survival was evaluated using Kaplan–Meier analysis with log-rank testing.

Candidate variables for the primary multivariable model were selected a priori based on clinical relevance, established circuit-level factors associated with filter patency, and previous literature [26–28], rather than solely on univariable P values. The primary Cox model included vascular access site, CRRT modality, dilution strategy, filter type, and filtration fraction. The model estimated the association between anticoagulant strategy and circuit-related filter failure conditional on these measured circuit-level characteristics. Patient-level cluster-robust standard errors were used to account for repeated circuits within patients, and a shared-frailty Cox model with a patient-level random effect was fitted as a sensitivity analysis.

An extended Cox model additionally included APACHE II score, vasopressor use, liver disease, platelet count, INR, and fibrinogen. These variables were selected to represent measured baseline illness severity, hepatic dysfunction, and coagulation status while limiting model complexity given the number of patients and events. The extended model evaluated the association after additional adjustment for these measured patient-level characteristics. These variables were ascertained at ICU admission rather than immediately before CRRT initiation or first exposure to NM or LMWH. Additional clot-specific Cox analyses used clotting-related circuit failure as the event of interest. The circuit-adjusted and extended clot-specific models used the same covariate sets and patient-level cluster-robust standard errors as the corresponding primary analyses.

The proportional hazards assumption was evaluated using Schoenfeld residuals. Cox analyses used the full observed duration of each circuit without administrative censoring at 72 h, whereas Kaplan–Meier curves were displayed through 72 h, corresponding to the manufacturer-recommended routine replacement interval for the CRRT filters used in this study. Where non-proportionality was detected, the corresponding hazard ratios were interpreted as average associations over the observed follow-up rather than constant effects over time.

To account for competing circuit-termination events, a clustered Fine–Gray model treated circuit-related failure as the event of interest and planned and clinical-event-related terminations as competing events, with event-free circuits administratively censored at 72 h. A corresponding clot-specific competing-risk analysis treated clotting-related termination as the event of interest and all other termination categories as competing events. Cumulative incidence functions were compared using Gray’s test.

Propensity score matching (PSM) was performed at the patient level as an exploratory sensitivity analysis. Candidate baseline covariates were considered based on observed between-group differences and their clinical relevance to anticoagulant selection or circuit outcomes, while model complexity was limited in view of the small NM cohort. The final propensity score model included 14 patient-level variables: age, sex, smoking history, hypertension, liver disease, APACHE II score, vasopressor use, ICU admission category, lactate, platelet count, serum creatinine, total bilirubin, INR, and fibrinogen. Propensity scores were estimated using logistic regression. NM-treated patients were matched 1:1 to LMWH-treated patients using nearest-neighbor matching without replacement, targeting the average treatment effect in the treated (ATT), with a standardized caliper of 0.2. Covariate balance was assessed using standardized mean differences and a love plot. Matched circuit outcomes were analyzed using patient-level cluster-robust Cox regression. Because matching was performed at the patient level, it was not expected to eliminate imbalance in circuit-level CRRT prescription characteristics. To address unequal circuit contributions, an additional sensitivity analysis was restricted to the first CRRT circuit initiated in each matched patient. This analysis used Kaplan–Meier analysis, the log-rank test, and Cox regression with robust variance clustered by matched pair, using the same endpoint definition and censoring rules as the primary analysis.

All eligible patients and circuits identified during the predefined 13-month study period were included; therefore, no formal a priori sample-size calculation was performed. A two-sided P value < 0.05 was considered statistically significant. Analyses were performed using R version 4.3.3, with the survival package (version 3.8-3) for Cox regression, MatchIt (version 4.7.2) for PSM, and crrSC (version 1.1.2) for clustered Fine–Gray analysis.

### Use of artificial intelligence tools

OpenAI’s ChatGPT was used to assist with language editing, manuscript organization and refinement of wording. No AI tool was used to generate or analyze the original clinical dataset. All scientific content, statistical results, data interpretation and final wording were critically reviewed and verified by the authors. No AI tool was used as an author. The authors take full responsibility for the content of this manuscript.

## Results

### Study population

**Fig. 1:**
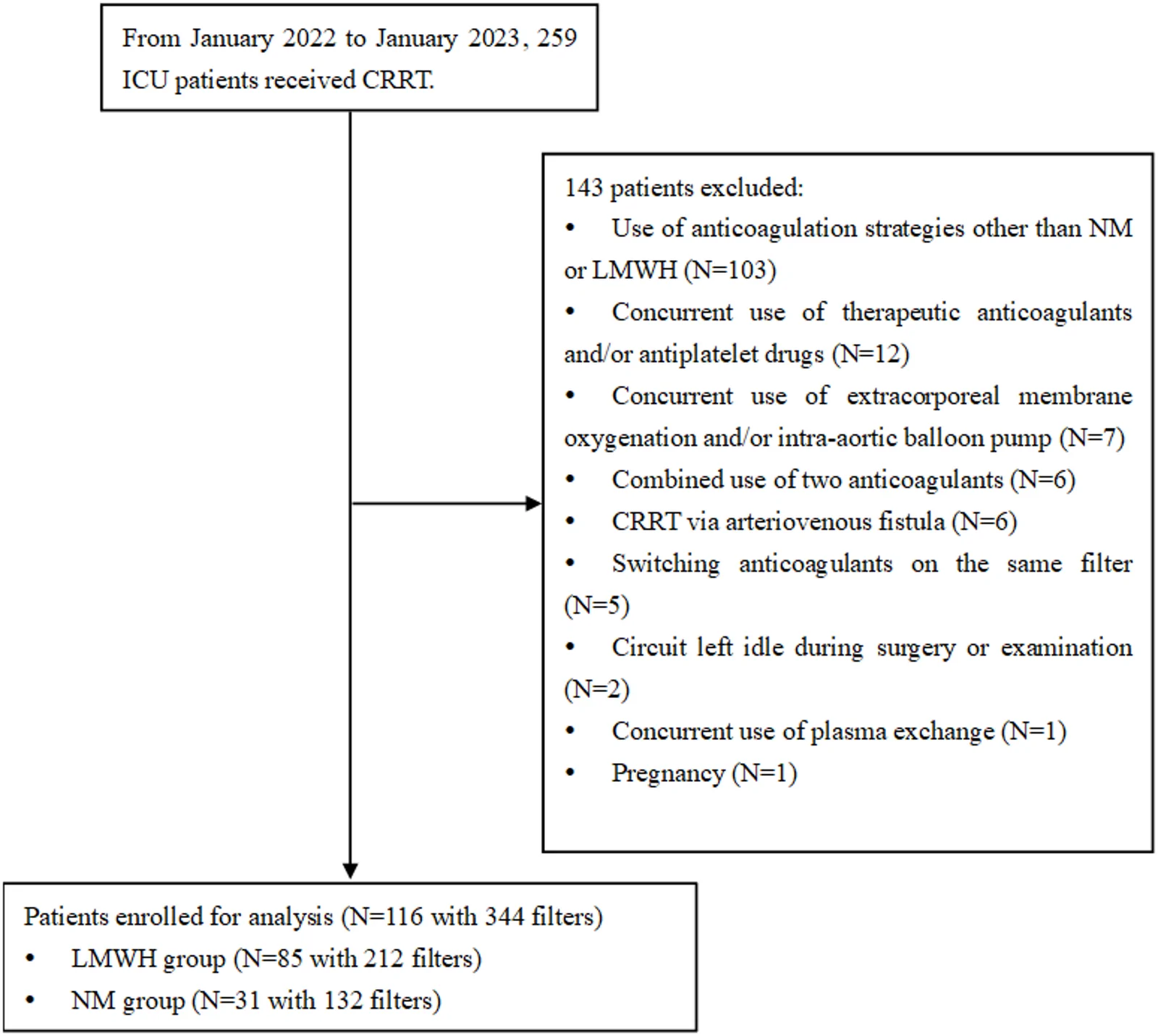
Study flow diagram. Flow diagram showing the screening and inclusion of ICU patients receiving CRRT from January 2022 to January 2023, including exclusion reasons and final allocation to the NM and LMWH groups

During the study period, 259 ICU patients received CRRT and were screened for eligibility. After applying the inclusion and exclusion criteria, 143 patients were excluded, mainly because they received anticoagulation strategies other than NM or LMWH, received concurrent therapeutic anticoagulation or extracorporeal support, or switched anticoagulants within the same circuit. Finally, 116 patients contributing 344 CRRT filters were included in the analysis: 31 patients with 132 filters in the NM group and 85 patients with 212 filters in the LMWH group (Fig. 1).

### Baseline characteristics

Baseline patient-level characteristics are summarized in Table 1. Compared with the LMWH group, patients in the NM group had a higher prevalence of liver disease (25.8% vs. 7.1%, *P* = 0.02), more frequent vasopressor use (61.3% vs. 31.8%, *P* = 0.01), and higher APACHE II scores (26.6 ± 10.4 vs. 21.3 ± 7.0, *P* = 0.002). Laboratory findings also suggested more pronounced coagulation and metabolic abnormalities in the NM group, including higher lactate levels [2.5 (1.3–5.8) vs. 1.3 (0.8–2.2) mmol/L, *P* < 0.001], higher total bilirubin levels [43.3 (26.5–158.4) vs. 18.8 (11.7–31.3) µmol/L, *P* < 0.001], prolonged prothrombin time [18.1 (15.6–23.7) vs. 16.5 (15.2–18.2) s, *P* = 0.04], higher INR [1.47 (1.32–2.08) vs. 1.34 (1.21–1.50), *P* = 0.01], lower platelet counts [106 (57–174) vs. 150 (107–256) ×10^9/L, *P* = 0.03], and lower fibrinogen levels [2.61 (1.89–3.79) vs. 4.18 (3.07–5.58) g/L, *P* < 0.001]. Serum creatinine was lower in the NM group than in the LMWH group [127 (92–287) vs. 270 (140–529) µmol/L, *P* = 0.03]. The distribution of ICU admission diagnoses and primary clinical bases for CRRT initiation did not differ significantly between groups. Detailed ICU admission categories are shown in Supplementary Table S1.

**Table 1:** Baseline characteristics of the overall cohort before propensity score matching

Variable	NM group (*n* = 31)	LMWH group (*n* = 85)	*P* value
**Demographics**			
Male sex, n (%)	24 (77.4)	61 (71.8)	0.71
Age, median (IQR), years	59 (48–70)	66 (54–79)	0.06
Height, median (IQR), cm	165 (163–168)	165 (160–168)	0.37
Weight, median (IQR), kg	60 (56–69)	60 (53–69)	0.66
Smoking history, n (%)	13 (41.9)	37 (43.5)	1.00
Comorbidities*, n (%)			
Hypertension	15 (48.4)	63 (74.1)	0.02
Diabetes mellitus	9 (29.0)	30 (35.3)	0.68
Cardiovascular disease	6 (19.4)	25 (29.4)	0.40
Liver disease	8 (25.8)	6 (7.1)	0.02
Chronic kidney disease	8 (25.8)	27 (31.8)	0.70
COPD	1 (3.2)	3 (3.5)	1.00
Stroke	3 (9.7)	9 (10.6)	1.00
Malignancy	12 (38.7)	26 (30.6)	0.55
Other comorbidities	9 (29.0)	29 (34.1)	0.77
**Mechanical ventilation, n (%)**	16 (51.6)	37 (43.5)	0.57
**Vasopressor use, n (%)**	19 (61.3)	27 (31.8)	0.01
**APACHE II, mean (SD)**	26.6 (10.4)	21.3 (7.0)	0.002
**Reason for ICU admission, n (%)**			0.17
Sepsis	12 (38.7)	20 (23.5)	
Postoperative	4 (12.9)	21 (24.7)	
Liver failure	2 (6.5)	2 (2.4)	
Other diagnoses	13 (41.9)	42 (49.4)	
**Primary clinical basis for CRRT initiation, n (%)**			0.83
KDIGO stage 3 with oliguria/anuria and progressive azotemia	23 (74.2)	56 (65.9)	
Fluid overload	0 (0.0)	1 (1.2)	
Heart failure	0 (0.0)	1 (1.2)	
Electrolyte imbalance	0 (0.0)	1 (1.2)	
Maintenance dialysis–dependent kidney failure	8 (25.8)	26 (30.6)	
**Time from ICU admission to CRRT initiation, median (IQR), days**	3.0 (1.2–7.7)	1.1 (0.5–5.1)	0.09
**Baseline laboratory values at ICU admission**			
Potassium, mean (SD), mmol/L	4.0 (0.6)	4.0 (0.7)	0.75
Hemoglobin, mean (SD), g/dL	9.6 (2.6)	9.2 (2.3)	0.41
Hematocrit, mean (SD)	29 (7)	28 (7)	0.53
pH, median (IQR)	7.35 (7.30–7.42)	7.39 (7.33–7.43)	0.07
Sodium, median (IQR), mmol/L	136 (133–141)	135 (131–138)	0.18
Ionized calcium, median (IQR), mmol/L	1.12 (1.08–1.17)	1.10 (1.02–1.15)	0.09
Lactate, median (IQR), mmol/L	2.5 (1.3–5.8)	1.3 (0.8–2.2)	< 0.001
Platelet count, median (IQR), ×10^9^/L	106 (57–174)	150 (107–256)	0.03
BUN, median (IQR), mmol/L	15.0 (10.7–20.5)	16.8 (12.6–23.8)	0.25
Serum creatinine, median (IQR), µmol/L	127 (92–287)	270 (140–529)	0.03
Total bilirubin, median (IQR), µmol/L	43.3 (26.5–158.4)	18.8 (11.7–31.3)	< 0.001
Prothrombin time, median (IQR), s	18.1 (15.6–23.7)	16.5 (15.2–18.2)	0.04
INR, median (IQR)	1.47 (1.32–2.08)	1.34 (1.21–1.50)	0.01
aPTT, median (IQR), s	47.4 (39.2–57.1)	44.1 (38.9–50.0)	0.22
Fibrinogen, median (IQR), g/L	2.61 (1.89–3.79)	4.18 (3.07–5.58)	< 0.001
D–dimer, median (IQR), mg/L	10.1 (3.5–13.1)	4.4 (2.5–10.1)	0.03
NT–proBNP, median (IQR), pg/mL	3013 (1162–14320)	5445 (1316–20150)	0.43

*Patients may have multiple comorbidities. Data are presented as mean (SD), median (IQR), or n (%). Abbreviations: APACHE II, Acute Physiology and Chronic Health Evaluation II; aPTT, activated partial thromboplastin time; ARDS, acute respiratory distress syndrome; BUN, blood urea nitrogen; COPD, chronic obstructive pulmonary disease; CRRT, continuous renal replacement therapy; ICU, intensive care unit; INR, international normalized ratio; IQR, interquartile range; KDIGO, Kidney Disease: Improving Global Outcomes; LMWH, low-molecular-weight heparin; NM, nafamostat mesylate; NT–proBNP, N–terminal pro–B–type natriuretic peptide; SD, standard deviation

After propensity score matching, 21 NM-treated patients were matched to 21 LMWH-treated patients. Matching reduced imbalance in several measured patient-level covariates, although residual imbalance remained; full SMDs are presented in Supplementary Table S2 and Supplementary Figure S1.

### CRRT prescription and circuit characteristics

CRRT circuit characteristics and treatment parameters are shown in Table 2. Vascular access site differed significantly between groups (*P* = 0.003). Right internal jugular access was more frequently used in the NM group than in the LMWH group (36.4% vs. 21.7%), whereas left femoral access was more common in the LMWH group (45.8% vs. 31.8%). CRRT modality also differed between groups (*P* < 0.001): CVVHDF was more common in the NM group (50.8% vs. 24.1%), whereas CVVHD was more common in the LMWH group (33.5% vs. 2.3%).

**Table 2:** Continuous renal replacement therapy circuit characteristics and treatment parameters in the overall filter cohort according to anticoagulation strategy

Variable	NM (132)	LMWH (212)	P value
**Vascular access site, n (%)**			0.003
Left femoral vein	42 (31.8)	97 (45.8)	
Left internal jugular vein	8 (6.1)	5 (2.4)	
Right femoral vein	34 (25.8)	64 (30.2)	
Right internal jugular vein	48 (36.4)	46 (21.7)	
**CRRT modality, n (%)**			< 0.001
CVVH	62 (47.0)	90 (42.5)	
CVVHD	3 (2.3)	71 (33.5)	
CVVHDF	67 (50.8)	51 (24.1)	
**Filter type, n (%)**			< 0.001
AV1000S	73 (55.3)	51 (24.1)	
M150	11 (8.3)	36 (17.0)	
oXiris	17 (12.9)	27 (12.7)	
ST150	31 (23.5)	98 (46.2)	
**Planned duration of CRRT, n (%)**			0.09
Less than 24 h	30 (22.7)	44 (20.8)	
At least 24 h to less than 48 h	70 (53.0)	93 (43.9)	
At least 48 h	32 (24.2)	75 (35.4)	
**Pre-dilution, n (%)**	43 (32.6)	3 (1.4)	< 0.001
**Filtration fraction, median (IQR), %**	20.9 (13.1–26.0)	23.0 (21.0–26.3)	< 0.001
**Reason for discontinuation of CRRT, n (%)**			0.49
Planned termination	63 (47.7)	107 (50.5)	
Clotting-related failure	50 (37.9)	68 (32.1)	
Access/device-related failure	13 (9.8)	20 (9.4)	
Clinical-event-related termination	6 (4.5)	17 (8.0)	
**TMP before circuit change, median (IQR), mmHg**	125 (100–151)	116 (50–158)	0.28

Reasons for CRRT discontinuation were categorized into four groups: planned termination, clotting-related failure, access/device-related failure, and clinical-event-related termination. Abbreviations: CRRT, continuous renal replacement therapy; NM, nafamostat mesylate; LMWH, low-molecular-weight heparin; CVVH, continuous venovenous hemofiltration; CVVHD, continuous venovenous hemodialysis; CVVHDF, continuous venovenous hemodiafiltration; IQR, interquartile range; TMP, transmembrane pressure

Filter type distribution differed significantly between groups (*P* < 0.001). AV1000S filters were used more frequently in the NM group than in the LMWH group (55.3% vs. 24.1%), whereas ST150 filters were more common in the LMWH group (46.2% vs. 23.5%). Pre-dilution was substantially more common in the NM group (32.6% vs. 1.4%, *P* < 0.001), whereas filtration fraction was higher in the LMWH group [23.0% (21.0–26.3) vs. 20.9% (13.1–26.0), *P* < 0.001]. Planned CRRT duration, grouped reasons for circuit discontinuation, and transmembrane pressure before circuit change were similar between groups. Detailed discontinuation reasons, observed filter lifespan, and actual anticoagulant exposure are provided in Supplementary Table S3. In NM-treated circuits, the median starting infusion rate was 8.5 mg/h (IQR 3.8–15.0), and the median cumulative dose per circuit was 130.0 mg (IQR 53.8–329.0). Among 817 available post-filter ACT measurements in NM-treated circuits, 668 (81.8%) were ≥ 160 s (Supplementary Table S3). In LMWH-treated circuits, the median initial dose was 4,000 IU (IQR 3,000–4,000), and the median cumulative dose per circuit was 8,000 IU (IQR 4,750–12,250).

In the propensity score–matched cohort, 150 circuits were analyzed: 105 from 21 NM-treated patients and 45 from 21 LMWH-treated patients. The NM group contributed a median of 3 circuits per patient (IQR 2–7; range 1–22), whereas the LMWH group contributed a median of 2 circuits per patient (IQR 1–3; range 1–5). Several circuit-level characteristics remained imbalanced between groups (Supplementary Table S4).

### Filter survival

**Fig. 2:**
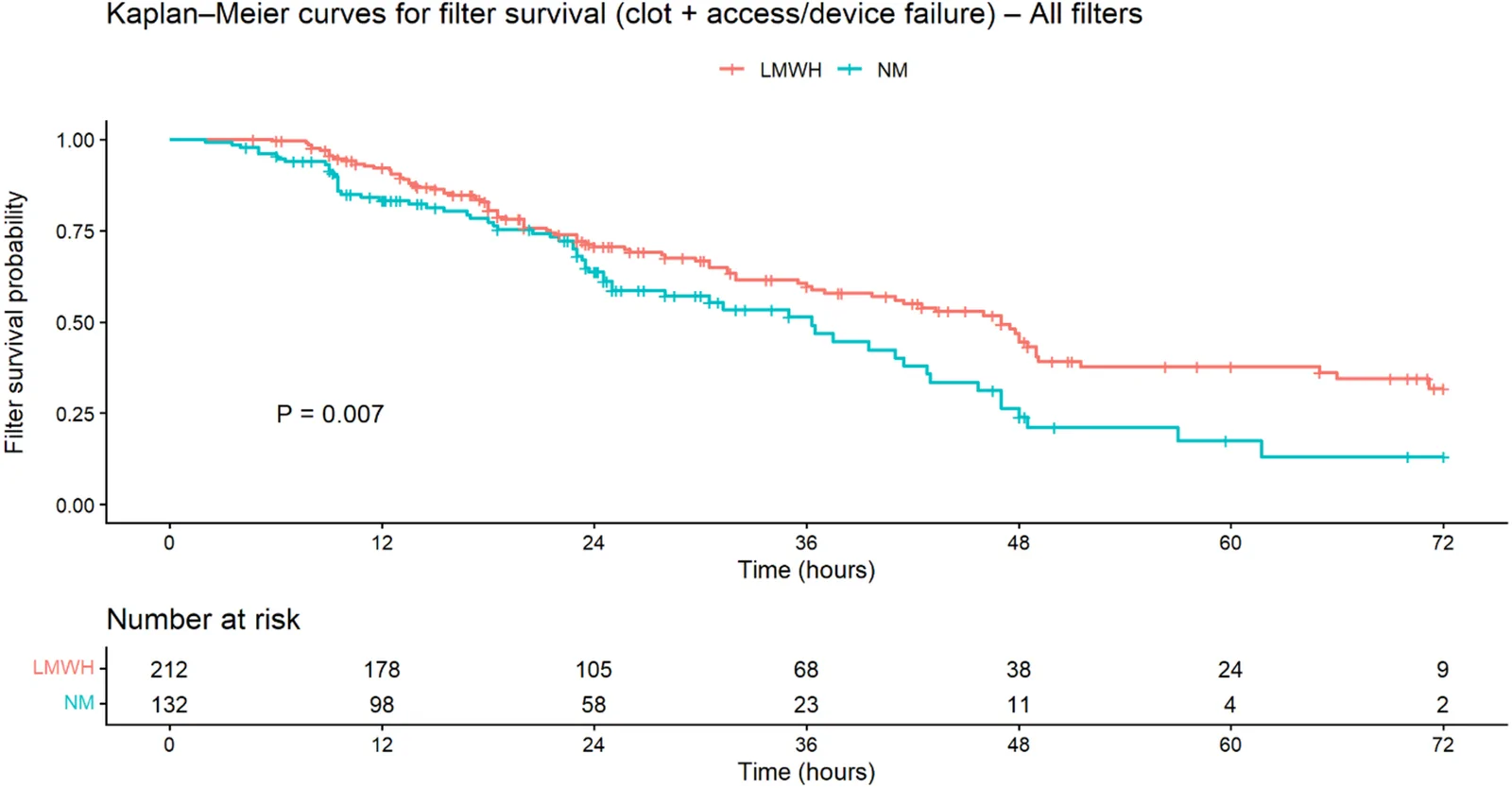
Kaplan-Meier curves for filter survival in the overall filter cohort. Filter survival was analyzed using the primary circuit-related failure definition, with clotting-related failure and access/device-related failure considered as events. Tick marks indicate censored observations. Events occurred in 63 of 132 NM-treated circuits and 88 of 212 LMWH-treated circuits. Numbers at risk are shown below the curves. The between-group comparison used the log-rank test. LMWH, low-molecular-weight heparin; NM, nafamostat mesylate. Alt text: Kaplan–Meier curves comparing filter survival between NM-treated and LMWH–treated CRRT circuits in the overall filter cohort under the primary circuit-related failure definition

Observed filter lifespan was shorter in the NM group than in the LMWH group [23.4 (11.7–30.0) vs. 29.7 (15.0–42.5) hours, *P* = 0.001; Supplementary Table S3]. In the primary time-to-event analysis, circuit-related failure occurred in 63 of 132 NM-treated circuits and 88 of 212 LMWH-treated circuits; Kaplan–Meier curves showed shorter unadjusted filter survival in the NM group (log-rank *P* = 0.007; Fig. 2). Under the clot-specific definition, 50 NM-treated and 68 LMWH-treated circuits experienced an event (log-rank *P* = 0.01; Supplementary Figure S2). Among circuits that experienced clotting-related failure, the median observed time to clotting was 21.8 h (IQR 9.6–29.9) in the NM group and 20.6 h (IQR 13.5–37.7) in the LMWH group (Supplementary Table S3). Under the non-planned termination definition, the corresponding event counts were 69 and 105 (log-rank *P* = 0.017; Supplementary Figure S3).

In the propensity score–matched cohort, differences in filter survival were not statistically significant under the primary circuit-related failure definition (Supplementary Figure S4) or the clot-specific failure definition (Supplementary Figure S5). In the first-circuit sensitivity analysis, circuit-related failure occurred in 10 of 21 NM-treated circuits and 11 of 21 LMWH-treated circuits. Median filter survival was 36.3 h in the NM group and 26.0 h in the LMWH group (log-rank P *=* 0.64; Supplementary Figure S6). A Cox model with robust variance clustered by matched pair yielded an imprecise estimate for the association between anticoagulant strategy and circuit-related failure (NM vs. LMWH: HR 0.81; 95% CI 0.32–2.06; *P* = 0.66; Supplementary Table S5).

### Multivariable Cox regression analyses

Results of the multivariable Cox regression analyses in the overall circuit cohort (344 circuits; 151 circuit-related failure events) are shown in Table 3. In the primary Cox model, after adjustment for vascular access site, CRRT modality, dilution strategy, filter type, and filtration fraction, anticoagulant strategy was not significantly associated with circuit-related filter failure (NM vs. LMWH: HR 1.41; 95% CI 0.88–2.25; *P* = 0.15). Similar results were observed in the shared-frailty Cox model with a patient-level random effect (HR 1.57; 95% CI 0.85–2.90; *P* = 0.15).

**Table 3:** Multivariable Cox regression analyses for circuit-related filter failure in the overall filter cohort

Variable	Category	Cluster-robust Cox HR (95% CI)	P value	Shared-frailty Cox HR (95% CI)	P value
Anticoagulant	LMWH (Ref)	—	—	—	—
	NM	1.41 (0.88–2.25)	0.15	1.57 (0.85–2.90)	0.15
Vascular access site	Right femoral vein (Ref)	—	—	—	—
	Left femoral vein	1.10 (0.71–1.72)	0.67	1.53 (0.87–2.67)	0.14
	Left internal jugular vein	0.72 (0.35–1.49)	0.38	1.19 (0.20–7.03)	0.85
	Right internal jugular vein	0.76 (0.37–1.54)	0.44	0.91 (0.39–2.15)	0.84
CRRT modality	CVVH (Ref)	—	—	—	—
	CVVHD	0.70 (0.33–1.48)	0.35	0.65 (0.31–1.40)	0.27
	CVVHDF	1.16 (0.71–1.90)	0.54	1.43 (0.79–2.58)	0.24
Dilution strategy	Post–dilution (Ref)	—	—	—	—
	Pre–dilution	0.21 (0.06–0.73)	0.02	0.15 (0.03–0.72)	0.02
Filter type	AV1000S (Ref)	—	—	—	—
	M150	0.34 (0.15–0.76)	0.01	0.25 (0.11–0.57)	< 0.001
	oXiris	0.82 (0.45–1.48)	0.51	0.70 (0.34–1.43)	0.33
	ST150	0.49 (0.31–0.77)	0.002	0.49 (0.28–0.88)	0.02
Filtration fraction	< 25% (Ref)	—	—	—	—
	≥ 25%	1.66 (1.03–2.68)	0.04	1.39 (0.78–2.49)	0.26

Circuit-related filter failure was defined as clotting-related failure or access/device-related failure; planned termination and clinical-event-related termination were treated as censored observations. The primary model was a Cox proportional hazards model with patient-level cluster-robust standard errors. The sensitivity model was a shared-frailty Cox model with a patient-level random effect. All variables shown in the table were included in the multivariable models. HRs > 1 indicate a higher hazard of circuit-related filter failure. CI, confidence interval; CRRT, continuous renal replacement therapy; CVVH, continuous venovenous hemofiltration; CVVHD, continuous venovenous hemodialysis; CVVHDF, continuous venovenous hemodiafiltration; HR, hazard ratio

Schoenfeld residual testing showed no evidence of non-proportionality for anticoagulant strategy (*P* = 0.83), and the global test was not statistically significant (*P* = 0.09). Evidence of non-proportionality was observed for filter type (*P* = 0.006) and filtration fraction (*P* = 0.004); the corresponding hazard ratios should therefore be interpreted as average associations over the observed follow-up rather than constant effects over time.

In the 72-hour clustered Fine–Gray sensitivity analysis of all 344 circuits, 151 circuit-related failure events occurred; the adjusted sHR for NM versus LMWH was 1.57 (95% CI 0.98–2.50; *P* = 0.06; Supplementary Table S6).

In the extended Cox model, which additionally included APACHE II score, vasopressor use, liver disease, platelet count, INR, and fibrinogen, anticoagulant strategy was not significantly associated with circuit-related failure (HR 1.16; 95% CI 0.70–1.90; *P* = 0.56; Supplementary Table S7).

In additional clot-specific Cox analyses, anticoagulant strategy was not significantly associated with clotting-related circuit failure after adjustment for the same circuit-level covariates as the primary model (NM vs. LMWH: HR 1.45; 95% CI 0.86–2.46; *P* = 0.16). In the extended clot-specific model additionally incorporating the measured patient-level covariates, the corresponding HR was 1.15 (95% CI 0.65–2.02; *P* = 0.63; Supplementary Table S8). In the clot-specific competing-risk analysis, the 72-hour cumulative incidence of clotting-related circuit failure was 37.9% in the NM group and 32.1% in the LMWH group (Gray’s test *P* = 0.22). In the adjusted clustered Fine–Gray model, the sHR for NM versus LMWH was 1.49 (95% CI 0.91–2.45; *P* = 0.11; Supplementary Table S8 and Supplementary Figure S7).

Several CRRT prescription factors were associated with circuit-related filter failure. Pre-dilution was associated with a lower hazard of failure in both the cluster-robust Cox model (HR 0.21; 95% CI 0.06–0.73; *P* = 0.02) and the shared-frailty Cox model (HR 0.15; 95% CI 0.03–0.72; *P* = 0.02). Compared with AV1000S filters, M150 filters were associated with lower hazards of circuit-related failure in both models (cluster-robust HR 0.34; 95% CI 0.15–0.76; *P* = 0.01; shared-frailty HR 0.25; 95% CI 0.11–0.57; *P* < 0.001). ST150 filters were also associated with lower hazards of failure (cluster-robust HR 0.49; 95% CI 0.31–0.77; *P* = 0.002; shared-frailty HR 0.49; 95% CI 0.28–0.88; *P* = 0.02). Filtration fraction ≥ 25% was associated with increased risk in the cluster-robust Cox model (HR 1.66; 95% CI 1.03–2.68; *P* = 0.04), although this association was attenuated and was not statistically significant in the shared-frailty model (HR 1.39; 95% CI 0.78–2.49; *P* = 0.26).

In the propensity score–matched cohort, circuit-related failure occurred in 48 of 105 NM-treated circuits and 24 of 45 LMWH-treated circuits. The adjusted Cox estimate was imprecise (NM vs. LMWH: HR 1.07; 95% CI 0.53–2.14; *P* = 0.85; Supplementary Table S9).

### Clinical outcomes and adverse events

Patient-level clinical outcomes and adverse events are summarized in Table 4. ICU length of stay [16.7 (6.6–27.3) vs. 10.0 (5.8–17.8) days, *P* = 0.06] and hospital length of stay [31.0 (18.0–67.5) vs. 24.0 (14.0–44.0) days, *P* = 0.11] did not differ significantly between the NM and LMWH groups. In-hospital mortality was 25.8% in the NM group and 17.6% in the LMWH group (*P* = 0.48), and 28-day mortality did not differ significantly between groups (38.7% vs. 40.0%, *P* = 1.00).

**Table 4:** Patient-level clinical outcomes and adverse events in the overall cohort

Variable	NM group (*n* = 31)	LMWH group (*n* = 85)	*P* value
**Clinical outcomes**			
ICU length of stay, median (IQR), days	16.7 (6.6–27.3)	10.0 (5.8–17.8)	0.06
Hospital length of stay, median (IQR), days	31.0 (18.0–67.5)	24.0 (14.0–44.0)	0.11
In–hospital mortality, n (%)	8 (25.8)	15 (17.6)	0.48
28–day mortality, n (%)	12 (38.7)	34 (40.0)	1.00
**Bleeding and transfusion outcomes**			
Bleeding events, n (%)	1 (3.2)	2 (2.4)	1.00
RBC transfusion, n (%)	20 (64.5)	30 (35.3)	0.009
**Acid-base and electrolyte disturbances and allergic reactions**			
Severe metabolic alkalosis, n (%)	5 (16.1)	9 (10.6)	0.63
Hyperkalemia, n (%)	2 (6.5)	4 (4.7)	0.66
Hypokalemia, n (%)	17 (54.8)	50 (58.8)	0.86
Hypercalcemia, n (%)	3 (9.7)	3 (3.5)	0.34
Severe hypocalcemia, n (%)	3 (9.7)	1 (1.2)	0.06
Metabolic acidosis, n (%)	0 (0.0)	2 (2.4)	1.00
Allergic reaction, n (%)	0 (0.0)	0 (0.0)	—

Data are presented as median (interquartile range) or n (%). Bleeding events were defined as clinically documented bleeding requiring transfusion of ≥ 2 units of red blood cells during CRRT or procedural or surgical hemostasis. Definitions of acid–base and electrolyte disturbances are provided in the Methods. P values were not calculated when no events occurred in either group. CRRT, continuous renal replacement therapy; ICU, intensive care unit; IQR, interquartile range; LMWH, low-molecular-weight heparin; NM, nafamostat mesylate; RBC, red blood cell

Clinically documented bleeding events were infrequent (3.2% in the NM group and 2.4% in the LMWH group; *P* = 1.00), precluding a precise between-group comparison of bleeding risk. However, RBC transfusion during CRRT was more frequent in the NM group than in the LMWH group (64.5% vs. 35.3%, *P* = 0.009). Acid-base and electrolyte disturbances showed no statistically significant between-group differences, although severe hypocalcemia was numerically more frequent in the NM group (9.7% vs. 1.2%, *P* = 0.06). No allergic reactions were documented in either group.

After propensity score matching, no significant between-group differences were observed in ICU or hospital length of stay, mortality, bleeding events, RBC transfusion, acid-base or electrolyte disturbances, or allergic reactions (Supplementary Table S10).

## Discussion

In this single-center observational cohort of adult ICU patients undergoing CRRT, unadjusted filter survival differed between the NM and LMWH groups, whereas no statistically detectable association between anticoagulant strategy and circuit-related filter failure was observed after adjustment for measured covariates. Across the adjusted and sensitivity analyses, estimates were imprecise and the confidence intervals remained compatible with clinically relevant differences in either direction, leaving the magnitude and direction of the comparative association uncertain.

The clinical relevance of directly comparing NM with LMWH should be considered in the context of their different patterns of use. LMWH is generally selected when systemic anticoagulation is acceptable [7, 10], whereas NM is often chosen when bleeding risk is perceived to be high or sustained systemic anticoagulation is undesirable [17, 20, 21]. However, “high bleeding risk” is not consistently defined across CRRT studies and clinical settings, with previous NM studies using heterogeneous criteria including thrombocytopenia, coagulation abnormalities, active bleeding, recent surgery, intracranial hemorrhage, septic shock, and disseminated intravascular coagulation [20, 21, 29]. These differences characterize the real-world clinical setting in which NM–LMWH comparisons arise, but also limit generalizability. NM use is concentrated largely in East Asia, whereas RCA and heparin-based strategies are more established internationally [6, 18, 30]. Accordingly, our findings are most relevant to settings where both NM and LMWH are used and should be extrapolated cautiously to regions with different anticoagulation practices.

These treatment-selection differences result in substantial confounding by indication. The NM group had greater illness severity, more frequent vasopressor use, more liver disease, and more pronounced coagulation abnormalities at baseline. Regression adjustment and propensity score matching can address measured differences but cannot eliminate confounding from unrecorded bleeding risk, dynamic clinical changes, or individualized treatment decisions. The attenuation of the unadjusted association after adjustment suggests that the crude difference in filter survival was at least partly related to measured patient- and circuit-level differences. Accordingly, the adjusted estimates should be interpreted as observational associations conditional on measured covariates rather than isolated treatment effects; their wide confidence intervals also preclude conclusions of equivalence.

Direct evidence comparing NM with LMWH remains limited, and the available pooled evidence should be interpreted in the context of substantial clinical and methodological heterogeneity. A recent meta-analysis restricted to CRRT patients at high bleeding risk found no significant overall difference in filter lifespan between NM and heparin, but heterogeneity was substantial (I² = 84.8%); notably, subgroup analyses yielded different estimates according to whether filter lifespan was summarized at the patient or filter level [30]. A broader systematic review and meta-analysis of blood-purification studies likewise found no significant overall difference in filter life between NM and conventional anticoagulants, while including heterogeneous populations, extracorporeal modalities, and comparator strategies [31]. These pooled estimates are therefore not directly comparable with the adjusted within-cohort association estimated here. The contributing studies differed in bleeding-risk profile, heparin comparator (UFH and/or LMWH), anticoagulant dosing, CRRT modality and prescription, filter characteristics, outcome definitions, and analytical approaches [30, 31]. Furthermore, most available comparisons were observational, and study-level pooled estimates cannot uniformly account for patient- and circuit-level confounding within individual cohorts.

The definition of circuit failure is also important. Overall circuit longevity and anticoagulant-specific efficacy are related but not identical constructs. The primary composite endpoint captures premature loss of a functioning circuit but is less specific to anticoagulation because some access/device failures arise predominantly from mechanical factors. Clotting-related failure therefore provides a more mechanistically specific endpoint. Previous NM–heparin studies primarily assessed overall filter lifespan or clot-free survival and differed in endpoint definitions, treatment protocols, observation windows, and handling of non-clotting circuit termination [19, 29–31]. In our cohort, the 72-hour cumulative incidence of clotting-related failure was 37.9% with NM and 32.1% with LMWH. Although the cumulative incidence and point estimates from the clot-specific Cox and competing-risk analyses were numerically higher with NM, the estimates were imprecise and compatible with clinically relevant differences in either direction. Thus, these analyses establish neither superiority nor equivalence and leave the relative association between anticoagulant strategy and clotting-related circuit failure uncertain.

The associations observed for CRRT prescription characteristics underscore the importance of considering circuit-level factors when interpreting filter survival. However, these secondary findings should be interpreted cautiously because several prescription characteristics were markedly imbalanced between treatment groups, and some associations were not consistent across analytical models. In addition, filter type and filtration fraction showed evidence of non-proportional hazards; their reported hazard ratios should therefore be interpreted as average associations over the observed follow-up rather than constant effects over time. Previous studies have likewise reported associations between circuit longevity and multiple non-pharmacologic factors, including hemoconcentration, membrane characteristics, vascular access, and operational settings [5, 26–28, 32–34]. Notably, observational and randomized evidence regarding individual circuit-management factors has not always been concordant [34, 35], reinforcing the need for cautious interpretation of such associations in non-randomized cohorts.

Clinically documented bleeding events were rare, with only three events observed in the entire cohort, precluding a reliable comparison of bleeding risk between NM and LMWH. RBC transfusion during CRRT was more frequent in the NM group; however, transfusion is a nonspecific outcome influenced by baseline anemia, surgery, illness severity, coagulopathy, and local transfusion practice and should not be interpreted as an anticoagulant-related bleeding endpoint. Accordingly, the present data are insufficient to draw comparative conclusions regarding the bleeding safety of NM and LMWH.

This study has several strengths. It directly compared NM and LMWH in adult ICU patients receiving CRRT, a comparison for which evidence remains scarce [29–31]. The analysis was performed at the circuit level while accounting for repeated circuits within patients, distinguished circuit-related failure from planned and clinical-event-related termination, and examined alternative event definitions and competing events. Adjustment for vascular access site, CRRT modality, dilution strategy, filter type, and filtration fraction allowed the observed association to be examined in the context of measured circuit-management characteristics, although residual circuit-level confounding cannot be excluded [5, 26–28]. Consecutive case inclusion and standardized data extraction were used to limit potential selection and information bias.

Several limitations should be acknowledged. First, the retrospective single-center design limits causal inference and generalizability. Anticoagulant selection was based on individualized clinical judgment, resulting in substantial confounding by indication. Although statistical adjustment can reduce measured imbalance, residual confounding cannot be excluded and comparative efficacy cannot be established. Moreover, patient-level covariates used for adjustment and matching were measured at ICU admission rather than immediately before CRRT or anticoagulant initiation; dynamic changes in vasopressor requirement, coagulation, liver function, and bleeding risk may therefore have introduced additional residual and time-varying confounding. Second, although starting and cumulative anticoagulant doses were available for all circuits, serial monitoring frequency, target attainment, and the reasons for dose adjustment, interruption, or discontinuation were not consistently available, limiting assessment of protocol heterogeneity. Third, no formal a priori sample-size calculation was performed, and the limited number of patients, particularly in the NM group, resulted in wide confidence intervals and limited statistical precision. Fourth, propensity score matching was performed at the patient level whereas the outcome was evaluated at the circuit level; residual patient- and circuit-level imbalance and unequal circuit contributions remained. The first-circuit sensitivity analysis removed repeated circuit contributions and yielded an HR of 0.81, but its wide confidence interval (95% CI 0.32–2.06) underscores the limited precision of estimates based on the small matched cohort. The matched analyses should therefore be considered exploratory. Fifth, planned and clinical-event-related terminations may have produced informative censoring; competing-risk analyses treated these terminations as competing events but remained statistically imprecise. Finally, safety events were infrequent, and detailed indications and timing of individual RBC transfusions were not consistently available.

## Conclusions

In this real-world ICU CRRT cohort, anticoagulant strategy was not significantly associated with circuit-related filter failure after adjustment for measured covariates. Because of substantial baseline differences, residual confounding, and limited statistical precision, the findings do not establish comparative efficacy, equivalence, noninferiority, or comparable safety. Larger prospective studies with standardized treatment and monitoring protocols are required.

## Supplementary Information

Below is the link to the electronic supplementary material.

Supplementary Material 1

## Data Availability

The datasets generated and analyzed during the current study are not publicly available because they contain de-identified but potentially sensitive clinical information from hospitalized patients. Data may be made available from the corresponding author upon reasonable request and with approval from the institutional ethics committee.

## Abbreviations

ACT: Activated clotting time
AKI: Acute kidney injury
APACHE II: Acute physiology and chronic health evaluation II
aPTT: activated partial thromboplastin time
BUN: Blood urea nitrogen
CI: Confidence interval
CRRT: Continuous renal replacement therapy
CVVH: Continuous venovenous hemofiltration
CVVHD: Continuous venovenous hemodialysis
CVVHDF: Continuous venovenous hemodiafiltration
HR: Hazard ratio
ICU: Intensive care unit
INR: International normalized ratio
IQR: Interquartile range
KDIGO: Kidney disease: improving global outcomes
LMWH: Low-molecular-weight heparin
NM: Nafamostat mesylate
NT-proBNP: N-terminal pro-B-type natriuretic peptide
PSM: Propensity score matching
RCA: Regional citrate anticoagulation
RBC: Red blood cell
SD: Standard deviation
TMP: Transmembrane pressure
UFH: Unfractionated heparin
