# Supplementary material for: Nafamostat mesylate versus low-molecular-weight heparin during continuous renal replacement therapy in critically Ill adults: a single-center retrospective cohort study
Source: BMC Nephrol. 2026 Sep 7;27:546. doi: 10.1186/s12882-026-05351-9 (PMC13587378; doi:10.1186/s12882-026-05351-9)
Supplement: Supplementary file 1 [file 12882_2026_5351_MOESM1_ESM.docx]

**Supplementary Appendix**

# Supplementary Methods

## Assessment of the proportional hazards assumption

The proportional hazards assumption for the primary multivariable Cox model was evaluated using Schoenfeld residuals. The assessment used the fixed-effect structure of the primary model, including anticoagulant strategy, vascular access site, CRRT modality, dilution strategy, filter type, and filtration fraction. Patient-level clustering was incorporated in the primary outcome model, whereas Schoenfeld residuals were used to assess whether individual fixed covariate effects varied over follow-up.

# Supplementary Results

## Proportional hazards assumption

Schoenfeld residual testing showed no evidence of non-proportionality for anticoagulant strategy (P = 0.834). The global Schoenfeld test was not statistically significant (P = 0.089). Evidence of non-proportionality was observed for filter type (P = 0.006) and filtration fraction (P = 0.004); the corresponding hazard ratios should therefore be interpreted as average associations over the observed follow-up rather than constant effects over time.

# Supplementary Tables

**Supplementary Table S1. Baseline characteristics of the overall cohort with detailed ICU admission categories**

| **Variable** | **NM group (n = 31)** | **LMWH group (n = 85)** | **P value** |
| --- | --- | --- | --- |
| **Demographics** | | | |
| Male sex, n (%) | 24 (77.4) | 61 (71.8) | 0.71 |
| Age, years, median (IQR) | 59 (48-70) | 66 (54-79) | 0.06 |
| Height, cm, median (IQR) | 165 (163-168) | 165 (160-168) | 0.37 |
| Weight, kg, median (IQR) | 60 (56-69) | 60 (53-69) | 0.66 |
| Smoking history, n (%) | 13 (41.9) | 37 (43.5) | 1.00 |
| **Comorbidities*, n (%)** | | | |
| Hypertension | 15 (48.4) | 63 (74.1) | 0.02 |
| Diabetes mellitus | 9 (29.0) | 30 (35.3) | 0.68 |
| Cardiovascular disease | 6 (19.4) | 25 (29.4) | 0.40 |
| Liver disease | 8 (25.8) | 6 (7.1) | 0.02 |
| Chronic kidney disease | 8 (25.8) | 27 (31.8) | 0.70 |
| COPD | 1 (3.2) | 3 (3.5) | 1.00 |
| Stroke | 3 (9.7) | 9 (10.6) | 1.00 |
| Malignancy | 12 (38.7) | 26 (30.6) | 0.55 |
| Other comorbidities | 9 (29.0) | 29 (34.1) | 0.77 |
| **Mechanical ventilation, n (%)** | 16 (51.6) | 37 (43.5) | 0.57 |
| **Vasopressor use, n (%)** | 19 (61.3) | 27 (31.8) | 0.01 |
| **APACHE II score, mean (SD)** | 26.6 (10.4) | 21.3 (7.0) | 0.002 |
| **Reason for ICU admission, n (%)** |  |  | 0.45 |
| Sepsis | 12 (38.7) | 20 (23.5) |  |
| Postoperative | 4 (12.9) | 21 (24.7) |  |
| Severe pneumonia/ARDS | 9 (29.0) | 16 (18.8) |  |
| Cardiac disease | 2 (6.5) | 10 (11.8) |  |
| Trauma | 1 (3.2) | 2 (2.4) |  |
| Acute kidney injury | 0 (0.0) | 4 (4.7) |  |
| Liver failure | 2 (6.5) | 2 (2.4) |  |
| Gastrointestinal disease | 0 (0.0) | 3 (3.5) |  |
| Neurological disease | 0 (0.0) | 3 (3.5) |  |
| Other diagnoses | 1 (3.2) | 4 (4.7) |  |
| **Primary clinical basis for CRRT initiation, n (%)** |  |  | 0.83 |
| KDIGO stage 3 with oliguria/anuria and progressive azotemia | 23 (74.2) | 56 (65.9) |  |
| Fluid overload | 0 (0.0) | 1 (1.2) |  |
| Heart failure | 0 (0.0) | 1 (1.2) |  |
| Electrolyte imbalance | 0 (0.0) | 1 (1.2) |  |
| Maintenance dialysis–dependent kidney failure | 8 (25.8) | 26 (30.6) |  |
| **Time from ICU admission to CRRT initiation, days, median (IQR)** | 3.0 (1.2-7.7) | 1.1 (0.5-5.1) | 0.09 |
| **Baseline laboratory values at ICU admission** | | | |
| Potassium, mmol/L, mean (SD) | 4.0 (0.6) | 4.0 (0.7) | 0.75 |
| Hemoglobin, g/dL, mean (SD) | 9.6 (2.6) | 9.2 (2.3) | 0.41 |
| Hematocrit, mean (SD) | 29 (7) | 28 (7) | 0.53 |
| pH, median (IQR) | 7.35 (7.30-7.42) | 7.39 (7.33-7.43) | 0.07 |
| Sodium, mmol/L, median (IQR) | 136 (133-141) | 135 (131-138) | 0.18 |
| Ionized calcium, mmol/L, median (IQR) | 1.12 (1.08-1.17) | 1.1 (1.02-1.15) | 0.09 |
| Lactate, mmol/L, median (IQR) | 2.5 (1.3-5.8) | 1.3 (0.8-2.2) | <0.001 |
| Platelet count, ×109/L, median (IQR) | 106 (57-174) | 150 (107-256) | 0.03 |
| BUN, mmol/L, median (IQR) | 15.0 (10.7-20.5) | 16.8 (12.6-23.8) | 0.25 |
| Serum creatinine, μmol/L, median (IQR) | 127 (92-287) | 270 (140-529) | 0.03 |
| Total bilirubin, μmol/L, median (IQR) | 43.3 (26.5-158.4) | 18.8 (11.7-31.3) | <0.001 |
| Prothrombin time, s, median (IQR) | 18.1 (15.6-23.7) | 16.5 (15.2-18.2) | 0.04 |
| INR, median (IQR) | 1.47 (1.32-2.08) | 1.34 (1.21-1.50) | 0.01 |
| aPTT, s, median (IQR) | 47.4 (39.2-57.1) | 44.1 (38.9-50.0) | 0.22 |
| Fibrinogen, g/L, median (IQR) | 2.61 (1.89-3.79) | 4.18 (3.07-5.58) | <0.001 |
| D-dimer, mg/L, median (IQR) | 10.1 (3.5-13.1) | 4.4 (2.5-10.1) | 0.03 |
| NT-proBNP, pg/mL, median (IQR) | 3013 (1162-14320) | 5445 (1316-20150) | 0.43 |

**Patients may have multiple comorbidities. Data are presented as mean (SD), median (IQR), or n (%). Abbreviation: APACHE II, Acute Physiology and Chronic Health Evaluation II; aPTT, activated partial thromboplastin time; ARDS, acute respiratory distress syndrome; BUN, blood urea nitrogen; COPD, chronic obstructive pulmonary disease; CRRT, continuous renal replacement therapy; ICU, intensive care unit; INR, international normalized ratio; IQR, interquartile range; KDIGO, Kidney Disease: Improving Global Outcomes; LMWH, low-molecular-weight heparin; NM, nafamostat mesylate; NT-proBNP, N-terminal pro-B-type natriuretic peptide; SD, standard deviation.*

**Supplementary Table S2. Baseline characteristics and covariate balance before and after propensity score matching**

| **Variable** | **NM group (n = 21)** | **LMWH group (n = 21)** | **SMD before matching** | **SMD after matching** |
| --- | --- | --- | --- | --- |
| **Demographics** | | | | |
| Male sex, n (%) | 17 (81.0) | 18 (85.7) | 0.13 | 0.13 |
| Age, years, median (IQR) | 59 (47-73) | 66 (47-77) | 0.36 | 0.15 |
| Height, cm, median (IQR) | 166 (165-168) | 165 (161-165) | 0.22 | 0.23 |
| Weight, kg, median (IQR) | 60 (60-70) | 60 (55-66) | 0.09 | 0.10 |
| Smoking history, n (%) | 9 (42.9) | 9 (42.9) | 0.03 | 0.00 |
| **Comorbidities*, n (%)** | | | | |
| Hypertension | 11 (52.4) | 14 (66.7) | 0.55 | 0.29 |
| Diabetes mellitus | 7 (33.3) | 6 (28.6) | 0.13 | 0.10 |
| Cardiovascular disease | 4 (19.0) | 7 (33.3) | 0.24 | 0.33 |
| Liver disease | 4 (19.0) | 3 (14.3) | 0.52 | 0.13 |
| Chronic kidney disease | 7 (33.3) | 7 (33.3) | 0.13 | 0.00 |
| COPD | 1 (4.8) | 1 (4.8) | 0.02 | 0.00 |
| Stroke | 1 (4.8) | 2 (9.5) | 0.03 | 0.19 |
| Malignancy | 9 (42.9) | 6 (28.6) | 0.17 | 0.30 |
| Other comorbidities | 8 (38.1) | 8 (38.1) | 0.11 | 0.00 |
| **Mechanical ventilation, n (%)** | 9 (42.9) | 11 (52.4) | 0.16 | 0.19 |
| **Vasopressor use, n (%)** | 11 (52.4) | 10 (47.6) | 0.62 | 0.10 |
| **APACHE II score, mean (SD)** | 25.0 (8.7) | 25.5 (8.2) | 0.60 | 0.06 |
| **Reason for ICU admission, n (%)** |  |  | 0.46 | 0.11 |
| Sepsis | 8 (38.1) | 7 (33.3) |  |  |
| Postoperative | 2 (9.5) | 2 (9.5) |  |  |
| Liver failure | 2 (9.5) | 2 (9.5) |  |  |
| Other diagnoses | 9 (42.9) | 10 (47.6) |  |  |
| **Primary clinical basis for CRRT initiation, n (%)** |  |  | 0.30 | 0.32 |
| KDIGO stage 3 with oliguria/anuria and progressive azotemia | 14 (66.7) | 14 (66.7) |  |  |
| Maintenance dialysis–dependent kidney failure | 7 (33.3) | 6 (28.6) |  |  |
| Electrolyte imbalance | 0 (0.0) | 1 (4.8) |  |  |
| **Time from ICU admission to CRRT initiation, days, median (IQR)** | 3.0 (1.9-7.8) | 2.8 (1.1-8.9) | 0.12 | 0.01 |
| **Baseline laboratory values at ICU admission** | | | | |
| Potassium, mmol/L, mean (SD) | 3.9 (0.6) | 4.0 (0.8) | 0.07 | 0.01 |
| Hemoglobin, g/dL, mean (SD) | 9.9 (2.7) | 8.7 (2.5) | 0.17 | 0.46 |
| Hematocrit, mean (SD) | 30 (8) | 27 (7) | 0.13 | 0.45 |
| pH, median (IQR) | 7.33 (7.29-7.41) | 7.39 (7.30-7.41) | 0.37 | 0.15 |
| Sodium, mmol/L, median (IQR) | 135 (133-141) | 135 (131-139) | 0.18 | 0.09 |
| Ionized calcium, mmol/L, median (IQR) | 1.14 (1.09-1.27) | 1.13 (1.05-1.14) | 0.34 | 0.29 |
| Lactate, mmol/L, median (IQR) | 1.6 (0.9-4.4) | 1.6 (0.9-5.0) | 0.68 | 0.12 |
| Platelet count, ×109/L, median (IQR) | 128 (76-283) | 129 (60-201) | 0.40 | 0.09 |
| BUN, mmol/L, median (IQR) | 15.0 (10.7-19.6) | 15.6 (9.50-21.4) | 0.16 | 0.05 |
| Serum creatinine, μmol/L, median (IQR) | 115 (79-249) | 175 (101-379) | 0.71 | 0.28 |
| Total bilirubin, μmol/L, median (IQR) | 41.3 (21.7-85.1) | 27.9 (12.4-43.3) | 0.75 | 0.03 |
| Prothrombin time, s, median (IQR) | 16.4 (15.2-22.0) | 17.6 (15.5-22.0) | 0.31 | 0.18 |
| INR, median (IQR) | 1.34 (1.29-1.98) | 1.42 (1.29-1.90) | 0.29 | 0.18 |
| aPTT, s, median (IQR) | 45.1 (38.6-50.2) | 45.1 (41.3-53.3) | 0.33 | 0.19 |
| Fibrinogen, g/L, median (IQR) | 3.08 (1.91-4.35) | 2.89 (1.87-5.17) | 0.76 | 0.04 |
| D-dimer, mg/L, median (IQR) | 10.1 (2.8-12.5) | 10.1 (4.4-16.9) | 0.33 | 0.36 |
| NT-proBNP, pg/mL, median (IQR) | 3414 (791-12702) | 3414 (710-17808) | 0.09 | 0.03 |

*Propensity scores were estimated using 14 baseline patient-level variables: age, sex, smoking history, hypertension, liver disease, APACHE II score, vasopressor use, ICU admission category, lactate, platelet count, serum creatinine, total bilirubin, INR, and fibrinogen. Absolute standardized mean differences are presented before and after matching; for multicategory variables, a single overall SMD is reported. *Patients may have multiple comorbidities. Data are presented as mean (SD), median (IQR), or n (%). Abbreviations: APACHE II, Acute Physiology and Chronic Health Evaluation II; aPTT, activated partial thromboplastin time; BUN, blood urea nitrogen; COPD, chronic obstructive pulmonary disease; CRRT, continuous renal replacement therapy; ICU, intensive care unit; INR, international normalized ratio; IQR, interquartile range; KDIGO, Kidney Disease: Improving Global Outcomes; LMWH, low-molecular-weight heparin; NM, nafamostat mesylate; NT-proBNP, N-terminal pro-B-type natriuretic peptide; SD, standard deviation; SMD, standardized mean difference.*

**Supplementary Table S3. Detailed CRRT circuit characteristics, discontinuation, and anticoagulant exposure according to anticoagulation strategy**

| **Variable** | **NM group (n = 132)** | **LMWH group (n = 212)** | **P value** |
| --- | --- | --- | --- |
| **Vascular access site, n (%)** |  |  | 0.003 |
| Left femoral vein | 42 (31.8) | 97 (45.8) |  |
| Left internal jugular vein | 8 (6.1) | 5 (2.4) |  |
| Right femoral vein | 34 (25.8) | 64 (30.2) |  |
| Right internal jugular vein | 48 (36.4) | 46 (21.7) |  |
| **CRRT modality, n (%)** |  |  | <0.001 |
| CVVH | 62 (47.0) | 90 (42.5) |  |
| CVVHD | 3 (2.3) | 71 (33.5) |  |
| CVVHDF | 67 (50.8) | 51 (24.1) |  |
| **Filter type, n (%)** |  |  | <0.001 |
| AV1000S | 73 (55.3) | 51 (24.1) |  |
| M150 | 11 (8.3) | 36 (17.0) |  |
| oXiris | 17 (12.9) | 27 (12.7) |  |
| ST150 | 31 (23.5) | 98 (46.2) |  |
| **Planned duration of CRRT, n (%)** |  |  | 0.09 |
| Less than 24 hours | 30 (22.7) | 44 (20.8) |  |
| At least 24 hours to less than 48 hours | 70 (53.0) | 93 (43.9) |  |
| At least 48 hours | 32 (24.2) | 75 (35.4) |  |
| **Pre-dilution, n (%)** | 43 (32.6) | 3 (1.4) | <0.001 |
| **Filtration fraction, median (IQR)** | 20.9 (13.1-26.0) | 23.0 (21.0-26.3) | <0.001 |
| **Anticoagulant exposure and monitoring** | | | |
| Starting NM infusion rate, mg/h, median (IQR) | 8.5 (3.8-15.0) | NA |  |
| Initial LMWH bolus, IU, median (IQR) | NA | 4000 (3000-4000) |  |
| Cumulative NM dose per circuit, mg, median (IQR) | 130.0 (53.8-329.0) | NA |  |
| Cumulative LMWH dose per circuit, IU, median (IQR) | NA | 8000 (4750-12250) |  |
| Post-filter ACT measurements, n | 817 | NA |  |
| Post-filter ACT ≥160 s, n (%) | 668 (81.8) | NA |  |
| **Reason for discontinuation of CRRT, n (%)** |  |  | 0.49 |
| Scheduled circuit change | 62 (47.0) | 105 (49.5) |  |
| Clot | 50 (37.9) | 68 (32.1) |  |
| Time to clotting among circuits with clotting-related failure, median (IQR), h | 21.8 (9.6–29.9) | 20.6 (13.5–37.7) | — |
| High TMP | 9 (6.8) | 16 (7.5) |  |
| Examination or operation | 3 (2.4) | 4 (1.9) |  |
| Catheter issues | 2 (1.5) | 3 (1.4) |  |
| High venous pressure | 2 (1.5) | 0 (0.0) |  |
| Discharge | 1 (0.8) | 11 (5.2) |  |
| Cardiac arrest | 1 (0.8) | 1 (0.5) |  |
| Death | 1 (0.8) | 1 (0.5) |  |
| Transfer to another department/unit | 1 (0.8) | 1 (0.5) |  |
| Change to an oXiris filter | 0 (0.0) | 1 (0.5) |  |
| Low TMP | 0 (0.0) | 1 (0.5) |  |
| **TMP before circuit change, median (IQR)** | 125 (100-151) | 116 (50-158) | 0.28 |
| **Filter lifespan, median (IQR), hours** | 23.4 (11.7-30.0) | 29.7 (15.0-42.5) | 0.001 |

Abbreviations: CRRT, continuous renal replacement therapy; NM, nafamostat mesylate; LMWH, low-molecular-weight heparin; CVVH, continuous venovenous hemofiltration; CVVHD, continuous venovenous hemodialysis; CVVHDF, continuous venovenous hemodiafiltration; IQR, interquartile range; TMP, transmembrane pressure; NA, not applicable. Detailed categories of CRRT discontinuation are presented instead of grouped classifications. Observed filter lifespan refers to the actual circuit duration from initiation to discontinuation, irrespective of the reason for discontinuation, and is presented as a descriptive, unadjusted comparison. Time to clotting was summarized descriptively only among circuits that experienced clotting-related failure and represents a conditional measure; it does not replace the full time-to-event analyses that included all circuits. No between-group hypothesis test was performed for this descriptive measure. Cumulative dose was calculated as the total amount of anticoagulant administered during each CRRT circuit. NM and LMWH exposure measures were presented separately and were not directly compared because the agents were administered using different regimens and recorded in different units.

**Supplementary Table S4. Circuit-level characteristics in the propensity score–matched cohort**

| **Variable** | **NM group (n = 105)** | **LMWH group (n = 45)** | **P value** |
| --- | --- | --- | --- |
| **Vascular access site, n (%)** |  |  | <0.001 |
| Left femoral vein | 30 (28.6) | 26 (57.8) |  |
| Left internal jugular vein | 8 (7.6) | 1 (2.2) |  |
| Right femoral vein | 22 (21.0) | 11 (24.4) |  |
| Right internal jugular vein | 45 (42.9) | 7 (15.6) |  |
| **CRRT modality, n (%)** |  |  | <0.001 |
| CVVHDF | 58 (55.2) | 17 (37.8) |  |
| CVVH | 46 (43.8) | 20 (44.4) |  |
| CVVHD | 1 (1.0) | 8 (17.8) |  |
| **Filter type, n (%)** |  |  | <0.001 |
| AV1000S | 69 (65.7) | 17 (37.8) |  |
| ST150 | 29 (27.6) | 12 (26.7) |  |
| oXiris | 6 (5.7) | 9 (20.0) |  |
| M150 | 1 (1.0) | 7 (15.6) |  |
| **Planned duration of CRRT, n (%)** |  |  | 0.61 |
| Less than 24 hours | 26 (24.8) | 12 (26.7) |  |
| At least 24 hours to less than 48 hours | 52 (49.5) | 21 (46.7) |  |
| At least 48 hours | 27 (25.7) | 12 (26.7) |  |
| **Pre-dilution, n (%)** | 43 (41.0) | 1 (2.2) | <0.001 |
| Filtration fraction, median (IQR) | 15.1 (13.1-27.2) | 22.0 (15.1-26.0) | 0.99 |
| **Reason for discontinuation of CRRT, n (%)** |  |  | <0.001 |
| Planned termination | 54 (51.4) | 16 (35.6) |  |
| Clotting-related failure | 39 (37.1) | 20 (44.4) |  |
| Access/device-related failure | 9 (8.6) | 4 (8.9) |  |
| Clinical-event-related termination | 3 (2.9) | 5 (11.1) |  |
| TMP before circuit change, median (IQR) | 106 (100-151) | 102 (60-154) | 0.20 |

Abbreviations: CRRT, continuous renal replacement therapy; NM, nafamostat mesylate; LMWH, low-molecular-weight heparin; CVVH, continuous venovenous hemofiltration; CVVHD, continuous venovenous hemodialysis; CVVHDF, continuous venovenous hemodiafiltration; IQR, interquartile range; TMP, transmembrane pressure. Reasons for CRRT discontinuation were categorized into four groups: planned termination, clotting-related failure, access/device-related failure, and clinical-event-related termination.

**Supplementary Table S5. Circuit contribution per patient and first-circuit sensitivity analysis in the propensity score–matched cohort**

| **Characteristic or outcome** | **NM group (n = 21)** | **LMWH group (n = 21)** | **HR (95% CI)** | **P value** |
| --- | --- | --- | --- | --- |
| **Matched cohort** | | | | |
| Patients, n | 21 | 21 |  |  |
| Total circuits, n | 105 | 45 | — |  |
| Circuits per patient, median (IQR) | 3 (2–7) | 2 (1–3) | — |  |
| Circuits per patient, range | 1–22 | 1–5 | — |  |
| **First-circuit sensitivity analysis** | | | | |
| Circuits analyzed, n | 21 | 21 |  |  |
| Circuit-related failure, n (%) | 10 (47.6) | 11 (52.4) | — |  |
| Median filter survival, h | 36.3 | 26.0 | — | 0.64 |
| NM versus LMWH | — | Reference | 0.81 (0.32–2.06) | 0.66 |

*The first circuit was defined as the CRRT circuit with the earliest initiation time for each matched patient. Circuit-related failure comprised clotting-related and access/device-related failure; other discontinuations were censored according to the primary analysis. The P value for median filter survival is from the log-rank test. The hazard ratio was estimated using a Cox model with robust variance clustered by matched pair. Abbreviations: CI, confidence interval; CRRT, continuous renal replacement therapy; HR, hazard ratio; IQR, interquartile range; LMWH, low-molecular-weight heparin; NM, nafamostat mesylate.*

**Supplementary Table S6. Clustered Fine–Gray competing-risk analysis for circuit-related filter failure in the overall cohort**

| **Outcome or model** | **NM group (n = 132)** | **LMWH group (n = 212)** | **sHR (95% CI)** | **P value** |
| --- | --- | --- | --- | --- |
| **Observed status within 72 h** | | | | |
| Circuit-related filter failure, n (%) | 63 (47.7) | 88 (41.5) | - |  |
| Planned termination, n (%) | 61 (46.2) | 101 (47.6) | - |  |
| Clinical-event-related termination, n (%) | 6 (4.5) | 17 (8.0) | - |  |
| Administrative censoring at 72 h, n (%) | 2 (1.5) | 6 (2.8) | - |  |
| **Clustered Fine-Gray models: NM versus LMWH** | | | | |
| Unadjusted model | - | Reference | 1.24 (0.65-2.36) | 0.52 |
| Adjusted model | - | Reference | 1.57 (0.98-2.50) | 0.061 |

*Circuit-related filter failure was defined as clotting-related or access/device-related failure. Planned termination and clinical-event-related termination were treated as competing events, and circuits remaining event-free beyond 72 h were administratively censored at 72 h. Subdistribution hazard ratios were estimated using clustered Fine-Gray regression with patient ID as the clustering variable. The adjusted model included anticoagulant strategy, vascular access site, CRRT modality, dilution strategy, filter type, and filtration fraction. CI, confidence interval; CRRT, continuous renal replacement therapy; LMWH, low-molecular-weight heparin; NM, nafamostat mesylate; sHR, subdistribution hazard ratio.*

**Supplementary Table S7. Extended patient-clustered Cox regression analysis for circuit-related filter failure in the overall cohort**

| **Variable** | **Adjusted HR (95% CI)** | **P value** |
| --- | --- | --- |
| Anticoagulant strategy: NM vs LMWH | 1.16 (0.70–1.90) | 0.56 |
| APACHE II score, per 5-point increase | 1.02 (0.88–1.18) | 0.81 |
| Vasopressor use: Yes vs no | 1.78 (1.13–2.81) | 0.01 |
| Liver disease: Yes vs no | 1.69 (0.78–3.65) | 0.18 |
| Platelet count, per 50 ×10^9/L increase | 1.00 (0.91–1.10) | 0.96 |
| INR, per 0.5-unit increase | 1.05 (0.97–1.14) | 0.18 |
| Fibrinogen, per 1-g/L increase | 0.96 (0.86–1.08) | 0.53 |

The model also included vascular access site, CRRT modality, dilution strategy, filter type, and filtration fraction. Patient-level cluster-robust standard errors were used. HR, hazard ratio; CI, confidence interval.

**Supplementary Table S8. Clot-specific Cox and competing-risk analyses of clotting-related circuit failure**

| **Analysis / model** | **NM group (n = 132)** | **LMWH group (n = 212)** | **Effect estimate** | **95% CI** | **P value** |
| --- | --- | --- | --- | --- | --- |
| **Panel A. Clot-specific Cox regression analyses** | | | | | |
| Circuit-adjusted Cox model, NM vs LMWH | — | — | HR 1.45 | 0.86–2.46 | 0.16 |
| Extended patient- and circuit-adjusted Cox model, NM vs LMWH | — | — | HR 1.15 | 0.65–2.02 | 0.63 |
| **Panel B. Clot-specific competing-risk analysis** | | | | | |
| 72-h cumulative incidence of clotting-related circuit failure | 37.9% | 32.1% | — | — | 0.22* |
| Adjusted clustered Fine–Gray model, NM vs LMWH | — | — | sHR 1.49 | 0.91–2.45 | 0.11 |

*Clot-specific Cox models treated only clotting-related circuit termination as the event of interest, with other termination categories censored. The circuit-adjusted model included vascular access site, CRRT modality, dilution strategy, filter type, and filtration fraction; the extended model additionally included APACHE II score, vasopressor use, liver disease, platelet count, INR, and fibrinogen. In the competing-risk analysis, access/device-related failure, planned termination, and clinical-event-related termination were treated as competing events. Circuits remaining event-free beyond 72 h were administratively censored at 72 h. The Fine–Gray model used the same circuit-level covariates as the circuit-adjusted Cox model and accounted for patient-level clustering. P value from Gray’s test. CI, confidence interval; HR, hazard ratio; LMWH, low-molecular-weight heparin; NM, nafamostat mesylate; sHR, subdistribution hazard ratio.

**Supplementary Table S9. Univariable and adjusted Cox regression analyses for circuit-related filter failure in the propensity score–matched cohort**

| **Variable** | **Category** | **Univariable HR (95% CI)** | **P value** | **Adjusted HR (95% CI)** | P value |
| --- | --- | --- | --- | --- | --- |
| Anticoagulant | LMWH (Ref) | — |  |  |  |
|  | NM | 0.73 (0.45–1.18) | 0.193 | 1.07 (0.53–2.14) | 0.852 |
| CRRT modality | CVVH (Ref) | — |  |  |  |
|  | CVVHD | 1.01 (0.24–4.23) | 0.986 | 1.14 (0.31–4.29) | 0.842 |
|  | CVVHDF | 0.76 (0.47–1.25) | 0.284 | 1.48 (0.72–3.05) | 0.284 |
| Dilution strategy | Post-dilution (Ref) | — |  |  |  |
|  | Pre-dilution | 0.23 (0.09–0.57) | 0.002 | 0.18 (0.05–0.66) | 0.01 |
| Filtration fraction | <25% (Ref) | — |  |  |  |
|  | ≥25% | 1.56 (0.98–2.47) | 0.06 | 1.20 (0.63–2.30) | 0.572 |

*Circuit-related filter failure was defined as clotting-related failure or access/device-related failure; planned termination and clinical-event-related termination were treated as censored observations. Adjusted HRs were estimated using a Cox proportional hazards model with patient-level cluster-robust variance estimation. Because the propensity score–matched cohort included fewer circuits and events, the adjusted model was restricted to a limited set of CRRT-related variables. These analyses should be interpreted as supplementary and exploratory. CI, confidence interval; CRRT, continuous renal replacement therapy; CVVH, continuous venovenous hemofiltration; CVVHD, continuous venovenous hemodialysis; CVVHDF, continuous venovenous hemodiafiltration; HR, hazard ratio.*

**Supplementary Table S10. Patient-level clinical outcomes and adverse events in the propensity score–matched cohort**

| **Variable** | **NM group (n = 21)** | **LMWH group (n = 21)** | **P value** |
| --- | --- | --- | --- |
| **Clinical outcomes** | | | |
| ICU length of stay, median (IQR), days | 23.0 (10.0–27.8) | 11.0 (9.3–23.0) | 0.15 |
| Hospital length of stay, median (IQR), days | 33.0 (19.0–69.0) | 20.0 (10.0–49.0) | 0.13 |
| In-hospital mortality, n (%) | 4 (19.0) | 5 (23.8) | 1.00 |
| 28-day mortality, n (%) | 7 (33.3) | 13 (61.9) | 0.12 |
| **Bleeding and transfusion outcomes** | | | |
| Bleeding events, n (%) | 1 (4.8) | 0 (0.0) | 1.00 |
| RBC transfusion, n (%) | 12 (57.1) | 5 (23.8) | 0.06 |
| **Acid–base and electrolyte disturbances and allergic reactions** | | | |
| Severe metabolic alkalosis, n (%) | 4 (19.0) | 0 (0.0) | 0.11 |
| Hyperkalemia, n (%) | 1 (4.8) | 0 (0.0) | 1.00 |
| Hypokalemia, n (%) | 11 (52.4) | 10 (47.6) | 1.00 |
| Hypercalcemia, n (%) | 3 (14.3) | 0 (0.0) | 0.23 |
| Severe hypocalcemia, n (%) | 0 (0.0) | 0 (0.0) |  |
| Metabolic acidosis, n (%) | 0 (0.0) | 0 (0.0) |  |
| Allergic reaction, n (%) | 0 (0.0) | 0 (0.0) |  |

*Data are presented as median (interquartile range) or n (%). Bleeding events were defined as clinically documented bleeding requiring transfusion of ≥2 units of red blood cells during CRRT or procedural or surgical hemostasis. Definitions of acid–base and electrolyte disturbances are provided in the Methods. P values were not calculated when no events occurred in either group. CRRT, continuous renal replacement therapy; ICU, intensive care unit; IQR, interquartile range; LMWH, low-molecular-weight heparin; NM, nafamostat mesylate; RBC, red blood cell.*

# Supplementary Figures

**Supplementary Figure S1. Covariate balance before and after propensity score matching.**

Absolute standardized mean differences are shown before and after matching. The dashed vertical line denotes 0.10.

**Supplementary Figure S2. Clot-specific filter survival in the overall circuit cohort.**

Only clotting-related circuit termination was treated as an event. Event counts were 50 of 132 circuits in the NM group and 68 of 212 circuits in the LMWH group.

**Supplementary Figure S3. Filter survival under the non-planned termination definition in the overall circuit cohort**

All non-planned terminations were treated as events and planned terminations were censored. Kaplan–Meier curves are displayed through 72 h. Event counts were 69 of 132 circuits in the NM group and 105 of 212 circuits in the LMWH group.

**Supplementary Figure S4. Filter survival in the propensity score–matched cohort using the primary circuit-related failure definition.**

Clotting-related and access/device-related failures were treated as events. Event counts were 48 of 105 circuits in the NM group and 24 of 45 circuits in the LMWH group.

**Supplementary Figure S5. Clot-specific filter survival in the propensity score–matched cohort.**

Only clotting-related termination was treated as an event. Event counts were 39 of 105 circuits in the NM group and 20 of 45 circuits in the LMWH group.

**Supplementary Figure S6. First-circuit filter survival in the propensity score–matched cohort.**

The analysis was restricted to the earliest CRRT circuit for each matched patient. Circuit-related failure occurred in 10 of 21 NM-treated circuits and 11 of 21 LMWH-treated circuits.

**Supplementary Figure S7. Cumulative incidence of clotting-related circuit failure according to anticoagulant strategy.**

Cumulative incidence functions for clotting-related circuit failure were estimated over 72 hours, with planned termination, access/device-related failure, and clinical-event-related termination treated as competing events. Circuits remaining in use beyond 72 hours were administratively censored at 72 hours. Gray’s test P = 0.224. LMWH, low-molecular-weight heparin; NM, nafamostat mesylate.
